# Spatial Integration of Somatosensory Inputs during Sensory-Motor Plasticity Phenomena Is Normal in Focal Hand Dystonia

**DOI:** 10.1155/2018/4135708

**Published:** 2018-10-10

**Authors:** C. Terranova, V. Rizzo, F. Morgante, R. Maggio, A. Calamuneri, G. Chillemi, P. Girlanda, A. Quartarone

**Affiliations:** ^1^Department of Clinical and Experimental Medicine, University of Messina, Messina, Italy; ^2^Department of Neurology, Humanitas Research Hospital, Rozzano, Milan, Italy; ^3^IRCCS Centro Neurolesi “Bonino Pulejo”, Messina, Italy; ^4^Department of Biomedical, Dental Science and Morphological and Functional Images, University of Messina, Italy

## Abstract

**Background:**

Surround inhibition is a system that sharpens sensation by creating an inhibitory zone around the central core of activation. In the motor system, this mechanism probably contributes to the selection of voluntary movements, and it seems to be lost in dystonia*. Objectives.* To explore if sensory information is abnormally processed and integrated in focal hand dystonia (FHD) and if surround inhibition phenomena are operating during sensory-motor plasticity and somatosensory integration in normal humans and in patients with FHD*. Methods.* We looked at the MEP facilitation obtained after 5 Hz repetitive paired associative stimulation of median (PAS M), ulnar (PAS U), and median + ulnar nerve (PAS MU) stimulation in 8 normal subjects and 8 FHD. We evaluated the ratio MU/(M + U) ∗ 100 and the spatial and temporal somatosensory integration recording the somatosensory evoked potentials (SEPs) evoked by a dual nerve input.

**Results:**

FHD had two main abnormalities: first, the amount of facilitation was larger than normal subjects; second, the spatial specificity was lost. The MU/(M + U) ∗ 100 ratio was similar in healthy subjects and in FHD patients, and the somatosensory integration was normal in this subset of patients*. Conclusions.* The inhibitory integration of somatosensory inputs and the somatosensory inhibition are normal in patients with focal dystonia as well as lateral surrounding inhibition phenomena during sensory-motor plasticity in FHD.

## 1. Introduction

Dystonia is a motor disorder characterized by sustained involuntary muscular contractions resulting from cocontraction of antagonistic muscles and overflow into extraneous muscles [1]. Focal hand dystonia frequently develops after repetitive movements in the presence of overtraining. These clinical observations have pointed out toward the presence of subtle abnormalities of plasticity, within somatosensory system, which may predispose individual to dystonia after excessive training [2]. Surround inhibition is a physiological mechanism to focus neuronal activity and to select appropriate neuronal responses and has been proposed to be an essential mechanism in the motor system, to sharp and focus motor activation [3, 4]. Surround inhibition (SI) can be tested in the motor system using TMS, and it has been demonstrated that this mechanism is deranged in patients with FHD [3].

In addition, it is well known that dystonia is characterized by a defective somatosensory processing within the somatosensory system [5] associated with a disturbance of sensorimotor integration [6–8]. Indeed, proprioceptive inputs coming from adjacent body parts are abnormally integrated in dystonia. This aberrant spatial gating, probably caused by an altered lateral surrounding inhibition, could contribute to the motor impairment present in dystonia [5]. Abnormalities of inhibition within the somatosensory system have been reported by Frasson et al. [9].

Several stimulation protocols can be used to test, noninvasively, plasticity within the somatosensory motor system. One of the most established protocols is the paired associative stimulation (PAS) where a magnetic stimulus is coupled with contralateral peripheral nerve stimulation [10]. This protocol exploits the principles of Hebbian LTP/LTD plasticity first described in animal experimentation. Patients with focal hand dystonia present two main alterations after PAS: first, the amount of facilitation is larger than normal; second and more important, the spatial specificity is lost so that facilitation also occurred in surrounding muscles [11]. PAS topographical specificity is probably related to inhibitory phenomena within motor cortex and is not related to a dual nerve simultaneously stimulation.

We have characterized a new conditioning fast PAS protocol that requires only two minutes of induction called 5 Hz rPAS [12] which produces plastic changes within both excitatory and inhibitory circuits within the sensory-motor cortex.

The aim of the present study was to evaluate the spatial integration of somatosensory inputs during sensory-motor plasticity phenomena, evaluated with 5 Hz rPAS, in healthy subjects and in patients with focal hand dystonia. To achieve this goal, we compared the amplitude of MEPs obtained after the 5 Hz rPAS protocol induced by stimulating the median and ulnar nerves simultaneously (MU) vs the MEP amplitude values being obtained from the arithmetic sum of the 5 Hz rPAS protocol elicited by stimulating the same nerves separately (M + U), looking at the amount of suppression induced by dual nerve simultaneously stimulation. Moreover, we evaluated the spatial and temporal somatosensory integration recording the somatosensory evoked potentials (SEPs) evoked by a dual nerve input to investigate the contribution of lateral inhibition in the somatosensory system. Indeed, previously, Tinazzi and coworkers proposed the MU/(M + U) ∗ 100 as a marker of lateral surround inhibition evoked by a dual input in the somatosensory system [5]. In that study, the increased ratio of SEP component elicited by median + ulnar stimulation indicated an abnormality of the intrinsic inhibitory interactions within the somatosensory system and hence a defect of lateral surround inhibition.

## 2. Materials and Methods

Eight patients with focal hand dystonia (6 male, 2 female, mean age 50.2 years) and 8 age- and sex-matched healthy subjects were recruited (see Table 1). Writer's cramp was classified as “simple” if dystonic features were present only with writing and as “dystonic” if muscle cramps also interfered with other motor tasks [13]. Participants did not receive any drug acting on the central nervous system and had no obvious history of neuropsychiatric diseases. All patients were tested at least 3 months after the last injections of botulinum toxin. All patients had normal structural MRI scans and did not show any mutation in the DYT1 gene. All subjects were right-handed according to the Edinburgh inventory. All subjects gave their informed consent, and the study was approved by the local ethics committee in accordance with the Declaration of Helsinki on the use of human subjects in experiments.

### 2.1. TMS and Recording Protocol

TMS was performed with a standard focal coil (mean loop diameter of 9 cm, Magstim Company, Whitland, Dyfed, UK). The coil was placed tangentially to the scalp at the optimum scalp position which consistently elicited the best motor evoked potentials (MEPs) in the right abductor pollicis brevis (APB) and abductor digiti minimi (ADM) muscles (“motor hot spot”).

### 2.2. Median and Ulnar Nerve Stimulation

Mixed electrical stimulation of the right median and ulnar nerves was performed at the wrist with the cathode located proximally. Peripheral stimulation was performed using a Digitimer D 160 stimulator (Digitimer, Welwyn Garden City, Herts, UK). The stimulus intensity was 200% of the perceptual threshold and the stimulus width 500 *μ*s.

### 2.3. Recording System

EMG was recorded from Ag-AgCl surface electrodes placed over the right abductor pollicis brevis (APB) and the right abductor digiti minimi (ADM) muscles using a belly-tendon montage. The signal was amplified and bandpass filtered (32 Hz to 1 KHz) by a DIGITIMER D 150 amplifier (Digitimer Ltd., Welwyn Garden City, Herts, UK) and stored at a sampling rate of 10 KHz (SigAvg Software, Cambridge Electronic Design, Cambridge, UK). EMG activity was continuously monitored, and trials in which the target will be not relaxed were excluded from analysis.

### 2.4. 5 Hz rPAS

The protocol consisted of 600 pairs of stimuli delivered at a rate of 5 Hz for two minutes. Each pair of stimuli included electrical peripheral nerve stimulation (CS) at 200% of the sensory threshold coupled with TMS at 90% active motor threshold over the motor hot spot. We take care of using always subthreshold intensities to avoid any muscle twitches produced by reafferent feedback during rPAS conditioning. The interstimulus interval (ISI) between the peripheral CS and the transcranial stimulus was fixed at 25 ms. Patients and controls received three different type of rPAS: rPAS median, rPAS ulnar, and rPAS median + ulnar. During MU rPAS, the stimulation site in the cortex was on the APB hotspot. The 5 Hz rPAS sessions were given in a random order, at least 1 week apart.

### 2.5. Measures of Cortical Excitability

We carefully monitored changes in cortical excitability after rPAS using single-pulse and paired-pulse TMS. The details of these techniques are given elsewhere. Several cortical excitatory parameters were taken into account before and after rPAS such as Resting Motor Threshold (RMT) and peak-to-peak MEP amplitude at rest. Measurements were acquired before 5 Hz rPAS (baseline), immediately after (T0), 15 minutes (T15), and 30 minutes (T30) after the end of the conditioning protocol. RMT is a well-standardized measure defined as the minimum intensity that could evoke a peak-to-peak MEP of 50 *μ*V in at least 5 out of 10 consecutive trials in the relaxed APB and ADM muscles [14]. In addition, we assess corticospinal excitability by collecting 20 consecutive MEPs from the motor hot spot of the APB and ADM muscles at a rate of 0.1 Hz. We tuned and adjusted the intensity of stimulation to obtain a MEP of ~1 mV in the target muscle. This intensity was kept constant throughout the experiment. In addition, for each muscle (APB and ADM), we evaluated the ratio MU/(M + U) ∗ 100, where MU is the MEP facilitation obtained after PAS with simultaneous stimulation of median and ulnar and M + U is the amount of MEP facilitation after PAS induced after stimulation of the individual nerves.

### 2.6. SEP Recording Procedure

SEP studies were conducted in a different day session in order not to interfere with PAS aftereffects. SEPs were obtained after stimulation of the median and the ulnar nerves at the wrist. Stimulation parameters were square pulses of 0.2 ms duration delivered at a rate of 2.2 Hz through Ag/AgCl surface electrodes (cathode proximal; impedance below 5 Kohm) over the nerve. Further details are reported elsewhere [5, 9]. Two different sessions were carried out. In the first session, where we assessed temporal somatosensory integration, the median nerve was stimulated with single stimuli (S1) and with paired stimuli (S1 + S2) at interstimulus intervals (ISIs) of 20 and 40 ms given in a random order. S2 (test stimulus) was obtained subtracting the S1 (control response) from the S1 + S2 (paired response). In the second session, where we examined spatial somatosensory integration, the median (M) and the ulnar (U) nerves were stimulated individually and simultaneously (MU). We averaged three hundred sweeps for each trial. Analysis time was fixed at 100 ms, and filtering bandwidth was set at 5–1500 Hz. SEPs were acquired using a Signal Software (Cambridge Electronic Design, Cambridge, UK). Cortical evoked response (N20) was derived from the parietal P3 scalp regions contralateral to the stimulation side and referred to the earlobe of the stimulated side. We measured peak-to-peak amplitudes and latencies at the peak of all SEPs. For the first session, we evaluated SEP amplitudes of control (S1) and test (S2) response and the amplitude ratio (S2/S1) ∗ 100 at 20 and 40 ms of ISIs. For the second session, we evaluated the ratio MU/(M + U) ∗ 100, where MU is the SEP amplitude produced from the concomitant stimulation of median and ulnar nerves, while M + U is the arithmetic sum of the SEPs originated by the stimulation of single nerve (For more details, see [5] and [9]).

### 2.7. Data Analysis

The effects of 5 Hz rPAS on RMT and peak-to-peak MEP amplitude were tested in separate repeated measure analysis of variance (ANOVA). For each dependent variable, we run a three-way repeated measure ANOVA with time (two levels: baseline and post), conditioning (three levels: PAS M, PAS U, and PAS MU) as within subject factor, and group (two levels: dystonia versus controls) as between subject factor. Conditional on a significant *P* value, post hoc *t*-tests were performed to investigate the strength of main effects and the patterns of interaction between factors. To evaluate the difference in the amount of surround inhibition after rPAS between focal dystonia and controls, we performed a factorial ANOVA. Moreover, to evaluate differences in SEP amplitude between dystonic patients and controls, we used the unpaired Mann Whitney *U* test.

A *P* value of <0.05 was considered significant. Data are given as mean ± standard error of the mean.

## 3. Results

**Figure 1:**
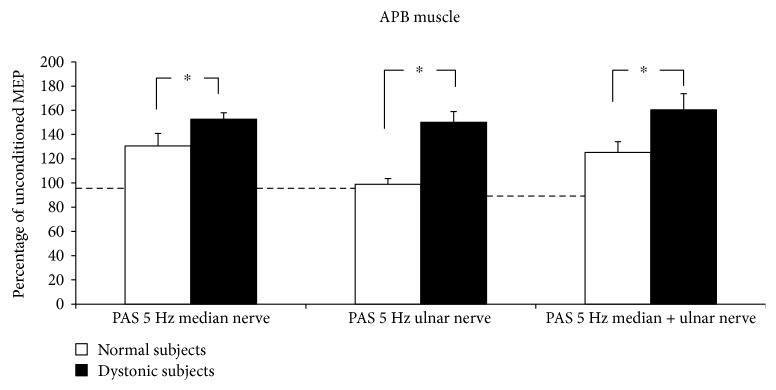
5 Hz rPAS induced an increase in MEP size recorded from APB muscle in both patients and controls; repeated measure ANOVA showed a significant effect of time [*F* = 88.38; *P* < 0.001], but the amount of facilitation was different between the two groups, as shown by the time × group interaction [*F* = 21.14; *P* < 0.001].

**Figure 2:**
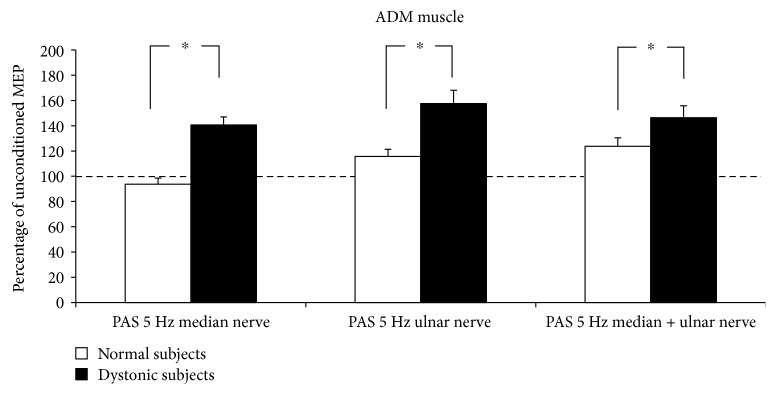
5 Hz rPAS induced an increase in MEP size recorded from ADM muscle in both patients and controls; repeated measure ANOVA showed a significant effect of time [*F* = 89.22; *P* < 0.001] and time × group interaction [*F* = 29.73; *P* < 0.001].

**Figure 3:**
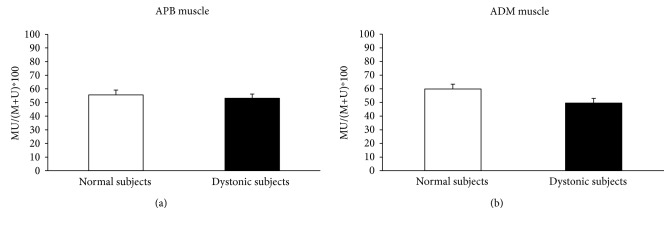
5 Hz rPAS did not induce any significant difference between the amount of ratio MU/(M + U) ∗ 100 after the 5 Hz rPAS between dystonic patients and controls in the APB and ADM muscles (factorial ANOVA APB: *F* = 0.596, *P* = 0.562 (a); ADM: *F* = 3.493, *P* = 0.07 (b)).

**Figure 4:**
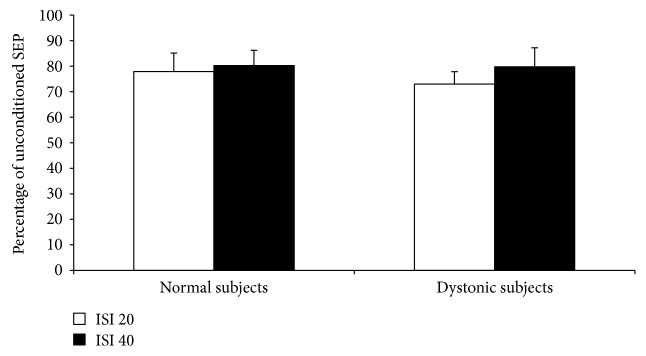
In normal subjects and focal dystonia patients, N20 SEP amplitudes of the S2 response were significantly suppressed at ISIs of 20 and 40 ms with respect to those of the S1 control response. The (S2/S1) ∗ 100 ratio of all central SEPs did not differ between patients and controls at the ISI of 20 and 40 ms (unpaired Mann Whitney *U* test: ISI 20 ms: *Z* = −0.4, *P* = 0.62; ISI 40 ms: *Z* = −0.9, *P* = 0.32).

**Figure 5:**
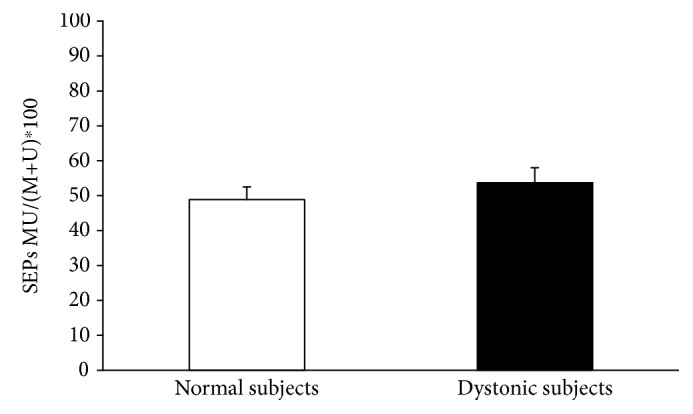
The MU/(M + U) ∗ 100 ratio of the cortical N20 SEP was not significantly different between dystonic patients and controls (unpaired Mann Whitney *U* test: *Z* = −0.2, *P* = 0.8).

5 Hz rPAS did not affect RMT either in controls or in dystonic patients as indexed by no effect of the factor time and group and intervention. Figures 1 and 2 plot differences in the amount of MEP facilitation, after 5 Hz rPAS, for the APB and ADM muscles, respectively, in patients and controls. 5 Hz rPAS increased MEP size recorded from APB muscle in both patients and controls; repeated measure ANOVA disclosed a significant effect of time [*F* = 88.38; *P* < 0.001], but the amount of facilitation was different between the two groups, as revealed by the time × group interaction [*F* = 21.14; *P* < 0.001]. This effect was produced by a larger increase in MEP amplitude in dystonic patients compared to controls. We found no time × group × conditioning interaction because all the three types of intervention induced an increase in MEP amplitude in both dystonic patients and controls [*F* = 1.51; *P* = 0.229] (Figure 1). Post hoc *t*-test revealed that in dystonic patients all the three types of intervention induced a significant increase in MEP amplitude [PAS M: *t* = −8.08, *P* < 0.001; PAS U: *t* = −5.1, *P* = 0.003; and PAS MU: *t* = −4.6, *P* = 0.007]. On the contrary, in controls, only PAS M and PAS MU induced changes in MEP amplitude but not PAS U [PAS M: *t* = −3.6, *P* = 0.008; PAS U: *t* = 0.3, *P* = 0.70; and PAS MU: *t* = −3.7, *P* = 0.007]. Similar statistical effects were observed in the ADM muscle: effect of time [*F* = 89.22, *P* < 0.001]; time × group interaction [*F* = 29.73, *P* < 0.001]; and time × group × conditioning interaction [*F* = 1.68, *P* = 0.19] (Figure 2). Post hoc *t*-test revealed again that in dystonic patients all the three types of intervention induced a significant increase in MEP facilitation [PAS M: *t* = −5.8, *P* = 0.001; PAS U: *t* = −5.7, *P* = 0.001; and PAS MU: *t* = −6.3, *P* < 0.001], while in controls, only PAS U and PAS MU induced changes in MEP amplitude but not PAS M [PAS M: *t* = 1.6, *P* = 0.15; PAS U: *t* = −2.7, *P* = 0.03; and PAS MU: *t* = −4.6, *P* = 0.002]. Factorial ANOVA did not show any significant difference between the amount of ratio MU/(M + U) ∗ 100 after the 5 Hz rPAS between dystonic patients and controls in the APB muscle. In both groups, indeed, the percentage of inhibition was around 50% [*F* = 0.596; *P* = 0.562] (Figure 3(a)). The same amount of inhibition was found in the ADM muscle for both patients and controls [*F* = 3.493; *P* = 0.07] (Figure 3(b)). In both normal subjects and focal dystonic patients, N20 SEP amplitudes of the S2 response were significantly inhibited at ISIs of 20 and 40 ms with respect to those of the S1 control response; more specifically, SEP amplitudes of the test S2 response were always smaller than those of the control S1 response. The (S2/S1) ∗ 100 ratio of all central SEPs did not differ between patients and controls at the ISI of 20 and 40 ms [ISI 20 ms: *Z* = −0.4, *P* = 0.62; ISI 40 ms: *Z* = −0.9, *P* = 0.32] (Figure 4). Finally, the MU/(M + U) ∗ 100 ratio of the cortical N20 SEP was not significantly different between dystonic patients and controls [*Z* = −0.2; *P* = 0.8] (Figure 5).

## 4. Discussion

Four main findings clearly emerge from this study:

1. All type of conditioning protocols (PAS M, PAS U, and PAS MU) can induce long-lasting plastic changes in cortical excitability of both dystonic patients and normal subjects
2. In keeping with previous findings, focal hand dystonia patients had two main abnormalities. First, associative plasticity after PAS25 was enhanced compared to normal subjects; second, the spatial specificity was lost so that facilitation was observed in both median and ulnar innervated muscles
3. The inhibitory integration of somatosensory inputs as well as the somatosensory inhibition are normal in patients with focal dystonia
4. Surround inhibition phenomena are normal in focal dystonia when applying PAS-induced sensory-motor plasticity protocol

### 4.1. PAS Aftereffects

Our results confirm that 5 Hz rPAS at an interstimulus interval of 25 ms can promote lasting changes in cortical excitability. Considering that rPAS aftereffects are long lasting, reversible, and topographically specific [12], this protocol is reminiscent of Hebbian plasticity models described in animal experimentation. A main advantage of rPAS protocol is the short duration of conditioning which makes this technique ideal to apply in patients [15]. In keeping with previous studies, we found a stronger increase in corticospinal excitability after rPAS in dystonic patients than in healthy controls. In addition, patients with dystonia showed loss of topographical specificity of PAS-induced effects, with facilitation spreading over median and ulnar innervated muscles, while in healthy individuals the increase in excitability only occurred in APB muscle innervated by the median nerve but not in the ADM muscle innervated by the ulnar nerve. The loss of spatial specificity is perhaps the most important and robust finding and could be related to the abnormalities of neuronal inhibition within motor cortex already identified in dystonic patients [16]. It is to point out that this excess of motor cortex plasticity is not confined to the clinically affected regions by dystonia but generalize across the entire sensorimotor system, representing an endophenotypic trait of the disease [17–20]. Although these findings have been reproduced by different groups [21–23], in one study, it has been reported that the effects of PAS were highly variable, and they conclude that enhanced plasticity should not be considered a dystonic fingerprint because the direction of response can vary, and there is an overlap between patient and healthy data [24].

### 4.2. Lateral Inhibition during PAS and within Somatosensory System

Inhibitory integration of somatosensory inputs as well as the somatosensory inhibition phenomena evaluated in our population of dystonic patients did not show any abnormalities compared with the ones of normal subjects. These findings support the idea that the temporal and spatial integration along somatosensory pathways are normal at least in focal hand dystonia, and this could explain the normal integration of the simultaneous median-ulnar nerve stimulation after PAS. On the other hand, our data confirm again that in focal hand dystonia there is a clear abnormality in sensory-motor plasticity as indexed by the loss of spatial specificity after PAS that may account for the creation of abnormal motor engrams. PAS topographical specificity has a different mechanism since the afferent stimulation is not dual and is probably related to the alteration of inhibitory phenomena within motor cortex which are lost in dystonia. These results can be apparently in contrast with the previous findings of Tinazzi and coworkers and Frasson and coworkers [5, 9]. Indeed, they found an abnormal somatosensory inhibition and sensory integration of afferent proprioceptive inputs. However, in both studies, the majority of patients were affected by generalized or segmental dystonia with only two patients having FHD [5, 9]. On the contrary, in our study, we only included a population affected by FHD. In a recent paper, Antelmi and coworkers [25] found a reduced suppression of SEPs in cervical dystonia at the ISI of 20 and 40 milliseconds, not confirmed in our study and in the study of Tamura and coworkers [26]. These contrasting results could be related to the fact that in the study of Antelmi and coworkers, SEPs were elicited by stimulation of the digital nerves of the index finger rather than the median nerve at the wrist. In the same paper, Antelmi and coworkers found an abnormal sensory integration in spatial domain in cervical dystonia, but again, this contrasting result might be due to the different methodology employed in the two studies [25].

### 4.3. Data Interpretation

In conclusion, the data of the present study suggest, in contrast with previous ones, that surround inhibition along somatosensory pathways are intact in FHD. In addition, we demonstrated that surround inhibition is also normal during the induction of sensory-motor plasticity phenomena. These results may suggest that, at least in FHD, spatial and temporal processing of sensory inputs are normal in patients with FHD despite the well-known alterations of spatial and temporal tactile discrimination, which are related to dysfunction in somatosensory cortex (S1) [26–29]. In a previous paper, Tamura and coworkers showed in FHD a reduction of inhibition of the P27 SEP component after a double stimulation of the median nerve at 5 ms interval, and they correlated this alteration with the abnormalities of temporal tactile discrimination. Similarly, the authors did not find any reduction of inhibition of N20 and P27 component at 20 and 40 ms intervals, as demonstrated in the present study [26]. On the other hand, Frasson and coworkers found a reduction of inhibition at 20 and 40 ms interval with a normalcy at 5 ms which was not confirmed by Tamura [9]. Therefore, future studies are needed to better clarify the link between the physiological mechanisms in tactile discrimination within S1 and the correlation with the cortical SEP components in healthy subjects and in the different form of dystonia. On the other hand, considering that spatial and temporal processing of sensory inputs are clearly abnormal in patients with generalized dystonia as demonstrated in the study of Tinazzi et al. and Frasson et al. [5, 9], we can speculate that a progressive loss of surround inhibition phenomena may contribute to the spreading and subsequent generalization of dystonia. Therefore, we can hypothesize that the greater is the spreading of dystonia in the body parts, the lesser is the ability to integrate and discriminate afferent sensory inputs coming simultaneously from adjacent body parts which could be subject of a subsequent study.

## Figures and Tables

**Table 1:** Clinical features.

Subjects	Age	Sex	Clinical features	Last botulinum toxin injection (months)	Patterns
1	50	M	Simple cramp	—	Predominant extensor pattern
2	55	M	Simple cramp	—	Predominant extensor pattern
3	31	F	Simple cramp	—	Predominant flexion pattern
4	62	M	Dystonic cramp	4	Predominant flexion pattern
5	38	M	Dystonic cramp	—	Predominant extensor pattern
6	66	M	Dystonic cramp	3	Predominant extensor pattern
7	55	F	Simple cramp	—	Predominant flexion pattern
8	45	M	Simple cramp	—	Predominant extensor pattern

## Data Availability

The data used to support the findings of this study are available from the corresponding author upon request.
